# The effect of peatland drainage and restoration on Odonata species richness and abundance

**DOI:** 10.1186/s12898-015-0042-z

**Published:** 2015-04-09

**Authors:** Merja Elo, Jouni Penttinen, Janne S Kotiaho

**Affiliations:** Department of Biological and Environmental Science, University of Jyväskylä, Jyväskylä, P.O.Box 35, 40014 Finland; Natural Heritage Service of Metsähallitus, Jyväskylä, P.O. Box 36, FIN-40101 Finland

**Keywords:** Ecological restoration, Disturbance, Mire, Dragonfly, Biodiversity

## Abstract

**Background:**

Restoration aims at reversing the trend of habitat degradation, the major threat to biodiversity. In Finland, more than half of the original peatland area has been drained, and during recent years, restoration of some of the drained peatlands has been accomplished. Short-term effects of the restoration on peatland hydrology, chemistry and vegetation are promising but little is known about how other species groups apart from vascular plants and bryophytes respond to restoration efforts.

**Results:**

Here, we studied how abundance and species richness of Odonata (dragonflies and damselflies) respond to restoration. We sampled larvae in three sites (restored, drained, pristine) on each of 12 different study areas. We sampled Odonata larvae before restoration (*n* = 12), during the first (*n* = 10) and the third (*n* = 7) year after restoration and used generalized linear mixed models to analyze the effect of restoration. Drained sites had lower abundance and species richness than pristine sites. During the third year after restoration both abundance and species richness had risen in restored sites.

**Conclusions:**

Our results show that Odonata suffer from drainage, but seem to benefit from peatland restoration and are able to colonize newly formed water pools already within three years after restoration.

**Electronic supplementary material:**

The online version of this article (doi:10.1186/s12898-015-0042-z) contains supplementary material, which is available to authorized users.

## Background

Degradation of natural habitats has become a major threat to biodiversity, questioning the survival of a vast number of species [1-3]. In addition to impoverishment of environment, the loss of species may compromise our own well-being as degradation of habitats may hamper ecosystem function [4], resulting even in ecosystem collapse [5]. In order to halt the loss of biodiversity and ecosystem services international targets have been set not only for slowing down the rate of degradation but also for restoring already degraded ecosystems [6]. In general, ecological restoration aims at reversing the degradation by partial or complete restoration of the original structure and function of the ecosystem [7].

Ecosystems with an urgent need for restoration are peatlands which have been, and still are, a subject for different human impacts, such as forestry, peat extraction and agriculture [8]. Peatlands cover approximately 3% of the Earth’s surface from which a majority, nearly 90%, is found in the northern hemisphere [8]. In Finland peatlands have been extensively drained, mainly for forestry purposes: over half of the original peatland area (altogether 4.7 million hectares) is currently drained [9]. This drastic diminishing of the natural peatland area has resulted in 223 species confined to peatlands classified as threatened [10].

Drainage leads to major and rapid changes in the hydrology and chemistry of peatland. Water level drops immediately by 20–60 cm [11-13] resulting in complex changes in the amount and availability of different nutrients [12-14], and in decrease of peat pH ([15] but see [13]). Consequently, these changes in abiotic conditions have their impact on the vegetation: peatland species confined to wet conditions are replaced by peatland species inhabiting hummocks and species colonizing from the nearby forests [15]. Peatland restoration aims at reversing these changes by damming or filling in the ditches with peat and by removing the trees grown after drainage [16]. Although studies concerning the long-term effects of these restoration efforts are still scarce (but see [12]) reported short-term effects occurring within a few years after restoration are promising: a rapid rise of the water-table and subsequent changes in peat chemistry [12,13,17,18], and also vegetation community seems to start recovering [16,19]. However, little is known how other species groups in addition to vascular plants and bryophytes respond to restoration of drained peatlands.

Odonata are widely used as bioindicators of different freshwater systems as they are sensitive to both local abiotic conditions and surrounding terrestrial landscape [20-26]. Particularly, Odonata have been used successfully in some restoration monitoring studies [27-30]. In Finland, there are altogether 55 species of Odonata and a handful of these species are restricted to peatlands (e.g. *Somatochlora arctica* and *S. alpestris*) [31]. In addition, peatlands are habitats also for generalists such as *Sympetrum danae* and *Libellulla quadrimaculata* occupying almost any kind of water [31]. Here we studied whether Odonata (dragonflies and damselflies) abundance and species richness is influences by drainage and whether they respond to peatland restoration already after three years. Since drainage is a severe disturbance that transforms the peatland habitat, it may be expected that drainage has had a negative impact on both the abundance and species richness of Odonata. At the short term, restoration may also be considered to be a disturbance and thus the short term impact of the restoration may be expected to be negative as well.

## Results

**Figure 1:**
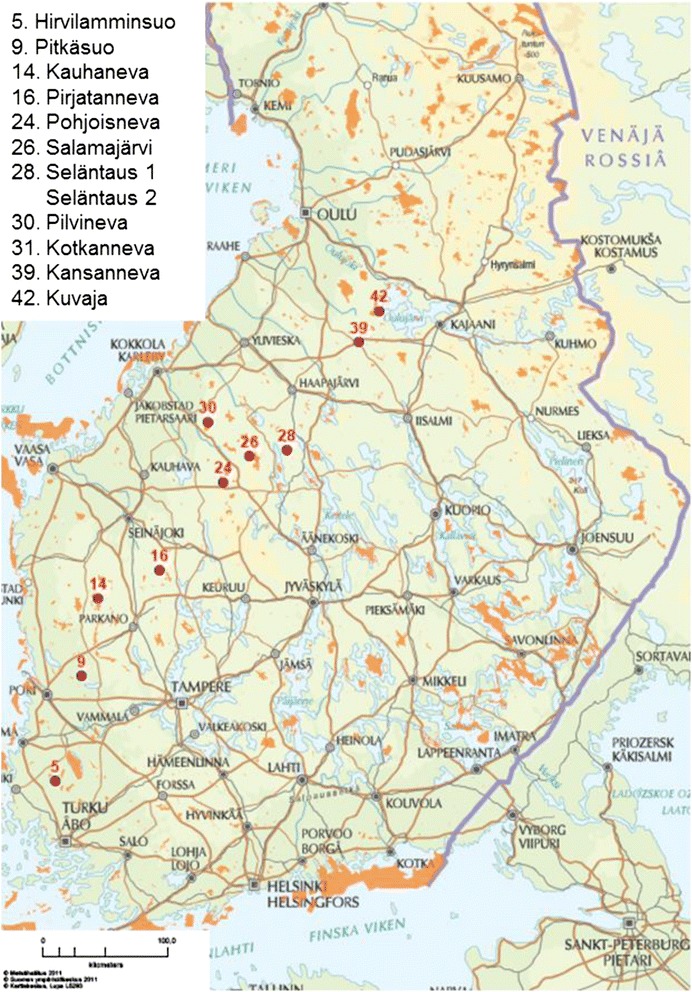
**Abundance of Odonata (mean and respective 95% Confidence Intervals) in restored, drained and pristine sites before restoration (black dots,** ***n*** **= 12), first year after restoration (white dots,** ***n*** **= 10) and third year after restoration (grey dots,** ***n*** **= 7).**

**Figure 2:**
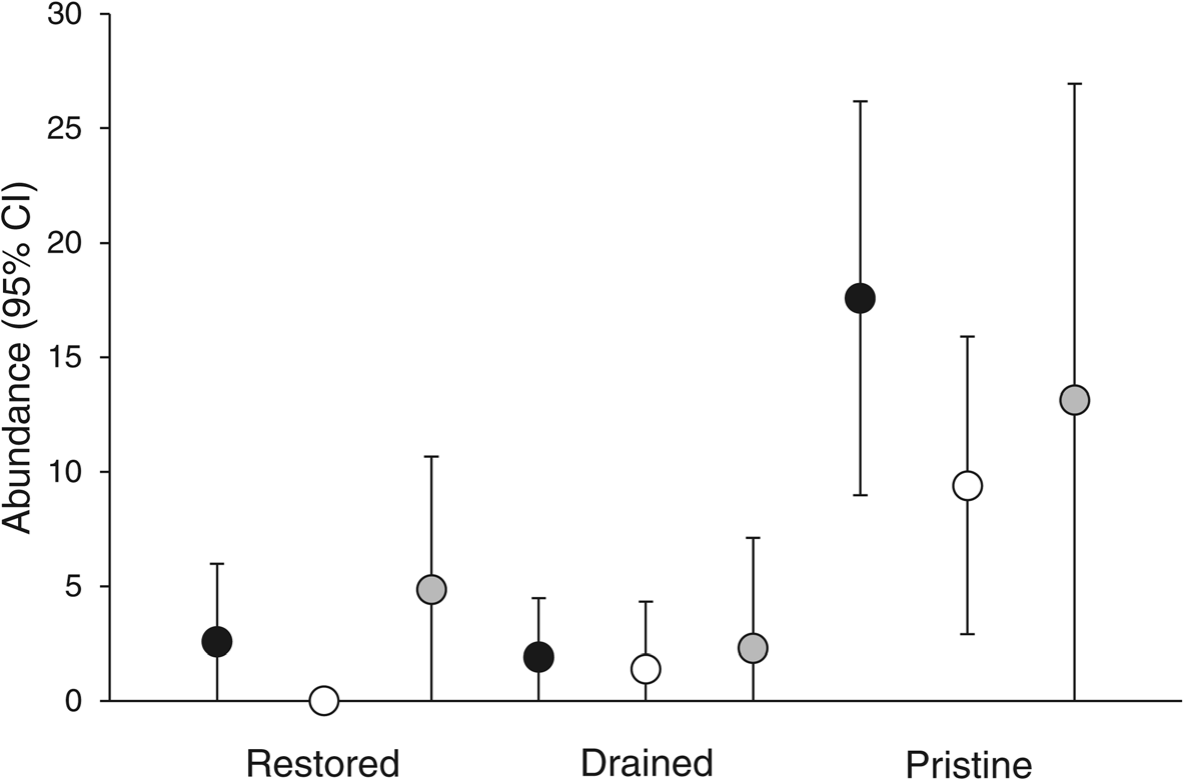
**Number of Odonata species (mean and respective 95% Confidence Intervals) in restored, drained and pristine sites before restoration (black dots,** ***n*** **= 12), first year after restoration (white dots,** ***n*** **= 10) and third year after restoration (grey dots,** ***n*** **= 7).**

We found altogether 515 individual larvae representing 13 species (Table 1). The number of individuals found from a site ranged from zero to 52 (mean = 5.9), and species richness from zero to five (mean = 1.1, when at least one individual was found: mean = 2.3). Both abundance and species richness was higher in pristine sites than in drained sites (Table 2). When considering also the first and the third year after restoration, interaction of treatment and year was statistically significant for both abundance and species richness: abundance and species richness of restored sites increases in the third year after restoration (Table 3, Figures 1 and 2). These results were consistent when analysing the data from the areas that had already been visited (n = 7) during the third year after restoration (Additional file 1: Table S2). The effect was due to three of the seven sites visited with relatively high species richness (4–5 species) whilst no individuals was found from the rest of the sites.

**Table 1:** **Odonata species found and the mean number of individuals of each species in different sites**

	**Restored**			**Drained**			**Pristine**		
	**Before**	**1. year after**	**3. year after**	**Before**	**1. year after**	**3. year after**	**Before**	**1. year after**	**3. year after**
*Lestes sponsa* (Hansemann, 1823)							2 (1)	2.5 (2)	3.7 (3)
*Coenagrion hastulatum* (Charpentier, 1825)			2 (1)				1.5 (2)	2.3 (3)	9 (2)
*Coenagrion johanssoni* (Wallengren, 1894)							6.5 (2)		
*Enallagma cyathigerum* (Charpentier, 1840)								2 (1)	
*Aeshna juncea* (Linnaeus, 1758)			1 (1)				2 (3)	1.3 (3)	1 (1)
*Aeshna subarctica* (Walker, 1908)			1 (1)			1 (1)	2.8 (5)	1 (1)	3 (1)
*Somatochlora alpestris* (Selys, 1840)			1 (1)	1 (1)					
*Somatochlora arctica* (Zetterstedt, 1840)	5.5 (4)			7.3 (3)	13 (1)	2.5 (2)	13 (1)		
*Somatochlora metallica* (Vander Linden, 1825)	1 (1)		2 (1)						
*Libellula quadrimaculata* (Linnaeus, 1758)			3.3 (3)				1.3 (3)	1 (1)	
*Sympetrum danae* (Sulzer, 1776)			1 (1)					1 (1)	8 (1)
*Leucorrhinia dubia* (Vander Linden, 1825)	8 (1)		2 (3)	1 (1)	1 (1)	10 (1)	16.8 (11)	8.9 (8)	9.6 (5)
*Leucorrhinia rubicunda* (Linnaeus, 1758)			5 (2)				1 (1)	1 (2)	1 (3)

In the parenthesis is the number of sites from where the species was caught.

Note that the number of sampled areas differs, before: *n* = 12; the first year after restoration: *n* = 10; the third year after restoration: *n* = 7.

**Table 2:** **Fixed effects part of generalized linear mixed models for abundance (a) and species richness (b) for all areas (** ***n*** **= 12) before restoration**

		**Estimate**	**SE**	**z**	***P***
a)	Intercept	**2.57**	**0.40**	**6.35**	**<0.001**
	treatment (D)	**−3.17**	**0.64**	**−4.98**	**<0.001**
	treatment (R)	**−2.90**	**0.61**	**−4.74**	**<0.001**
b)	Intercept	**0.88**	**0.19**	**4.75**	**<0.001**
	treatment (D)	**−1.98**	**0.53**	**−3.71**	**<0.001**
	treatment (R)	**−1.58**	**0.45**	**−3.51**	**<0.001**

Pristine sites are used as baselines, and treatment (D) = drained sites, treatment (R) = restored sites. Random variables = study area, study site; number of observations = 36, residual degrees of freedom = 31.

Number in bold indicate statistically significant results.

**Table 3:** **Fixed effects part (year, treatment, and their interaction term) of generalized linear mixed models for abundance (a) and species richness (b) for all areas (** ***n*** **= 12)**

		**Estimate**	**SE**	**z**	***cpb***
a)	Intercept	−0.77	0.62	−1.25	0.211
	year (1)	−18.53	1998.18	−0.01	0.993
	year (3)	**2.01**	**0.47**	**4.31**	**<0.001**
	treatment (D)	−0.57	0.82	−0.70	0.485
	treatment (R)	**3.38**	**0.71**	**4.79**	**<0.001**
	year (1):treatment (D)	18.05	1998.18	0.01	0.993
	year (3):treatment (D)	−1.14	0.62	−1.85	0.064
	year (1):treatment (R)	17.81	1998.18	0.01	0.993
	year (3):treatment (R)	**−1.96**	**0.49**	**−4.02**	**<0.001**
b)	Intercept	−0.83	0.45	−1.85	0.064
	year (1)	−19.15	248.36	−0.08	0.939
	year (3)	**1.40**	**0.50**	**2.78**	**0.006**
	treatment (D)	−0.41	0.66	−0.62	0.539
	treatment (R)	**1.62**	**0.48**	**3.39**	**0.001**
	year (1):treatment (D)	18.64	248.36	0.08	0.940
	year (3):treatment (D)	−0.88	0.87	−1.01	0.311
	year (1):treatment (R)	19.10	248.36	0.08	0.939
	year (3):treatment (R)	**−1.49**	**0.59**	**−2.51**	**0.012**

Before restoration and pristine sites are used as baselines. Year (1) = first year after restoration, year (3) = third year after restoration, treatment (D) = drained sites, treatment (R) = restored sites. Random variables = study area, study site; number of observations = 87, residual degrees of freedom = 76.

Number in bold indicate statistically significant results.

Inspecting SACs showed that where more than one species was found, SACs did not tend to reach an asymptote (Additional file 1: Figure S1). Moreover, there was variation between the curves between the years in some sites (e.g. Additional file 1: Figure S1d & g). Thus, the sampling could not be taken as exhaustive. In cases where an asymptote seems to be reached (e.g. Additional file 1: Figure S1i,j & f) the samples represent *Leucorrhinia dubia* which is commonly found in high abundance among the *Sphagnum* mosses. Comparing SACs among restored, drained and pristine sites was hindered by the fact that no individuals were found in many of the restored and drained sites. However, in the restored sites where individuals were found SACs lay above the curves of pristine sites suggesting that more species with the same number of individuals was found from the restored sites (Additional file 1: Figure S1a,c & l).

## Discussion

Our results showed that as expected Odonata clearly suffered from peatland drainage as both abundance and species richness of Odonata were lower in drained than in pristine sites. The reason for lower abundance and species richness in drained sites may be due to multiple factors: peatland drainage may change either the larval habitat or the surrounding landscape, and the effect may be direct or indirect *via* their prey. The simplest and the most probable cause for lower Odonata abundance in drained sites is the reduction of the available breeding habitat. Adult Odonata find suitable habitat by visual cues [32,33]. Thus, transformation from bog pools (with a cover ranging from several square meters to more than a hectare) to a ditch with an approximately meter wide water surface may significantly diminish the changes for finding the site or simply reduce its attractiveness. Moreover, due to their small size drifts are also susceptible for drying and they may also sustain smaller amount of other invertebrates, lowering the amount of prey for Odonata.

Drainage also changes water quality which may affect Odonata, either directly or indirectly *via* decreasing their prey. Particularly, drainage may result in decrease of pH [15]. Indeed, pH has been shown to be an important factor affecting Odonata community composition [34]. However, the suggested causality has been an indirect effect due to fish predation [35,36], and fish are unlikely to occur in bog pools. Although Odonata in general are relatively tolerant to low pH it may affect survival of some species [33]. In addition to water quality drainage results in inevitable changes in vegetation patterns [12,15]. This may affect Odonata as they use vegetation for multiple purposes such as for hunting or shelter [37], and a positive relationship between Odonata and plant species richness have been found at multiple spatial scales [38-40]. However, the fact that in some sites Odonata species respond rapidly to restoration suggests that vegetation patterns are not at least the main reason for diminished abundance in drained sites as the response of vegetation to restoration are generally rather slow [11,16,19].

As expected, during the first summer after restoration no larvae were found from the restored sites. This confirms that the increased abundance and species richness during the third year after restoration are due to new colonizations. Thus, Odonata are able to rapidly colonize the newly formed pools and seem to benefit from peatland restoration. However, increase of abundance and species richness was found only in three of the seven sites. By contrast, no individuals were found in four of the sites. This may be because just by chance the adults have not yet been able to find the pools. This may be unlikely however, because although landscape structure influences the movements of Odonata [41], the study was conducted on Natura 2000 sites and the set-up included large areas of pristine peatland very close in the same mire complex acting as a source pool. The other possible reasons that no individuals were found from some restored sites are that either the adults do not find the newly formed pools suitable for egg laying or larvae may still be in so low abundance that they were not detected.

In general the Odonata larvae were found in very low abundances from the studied peatlands. As the number of species increases with increasing number of individuals [42] and species accumulation curves representing this relationship failed to reach asymptotes, the sampling was not exhaustive enough to reveal the true Odonata species richness of the study sites. Thus, in order to have a better estimate of absolute species richness of the sites, sampling should have been enhanced. However, our main results are about the relative species richness and abundance among the pristine, drained and restored sites and for this comparison the sampling is robust enough.

## Conclusions

To conclude, Odonata showed decreased abundance and species richness in drained peatlands compared to pristine ones. Thus, drainage cause changes in hydrological and nutrient regimes in peatlands that have consequences not only to vegetation but also to other species groups. As the Odonata are top predators in many bog pools, their diminishing may cause cascading effects in other water invertebrates also. Fortunately, our results also showed that peatland restoration have potential to lead a relatively rapid recovery of Odonata abundance and species richness.

## Methods

### Study sites and sampling

**Figure 3:**
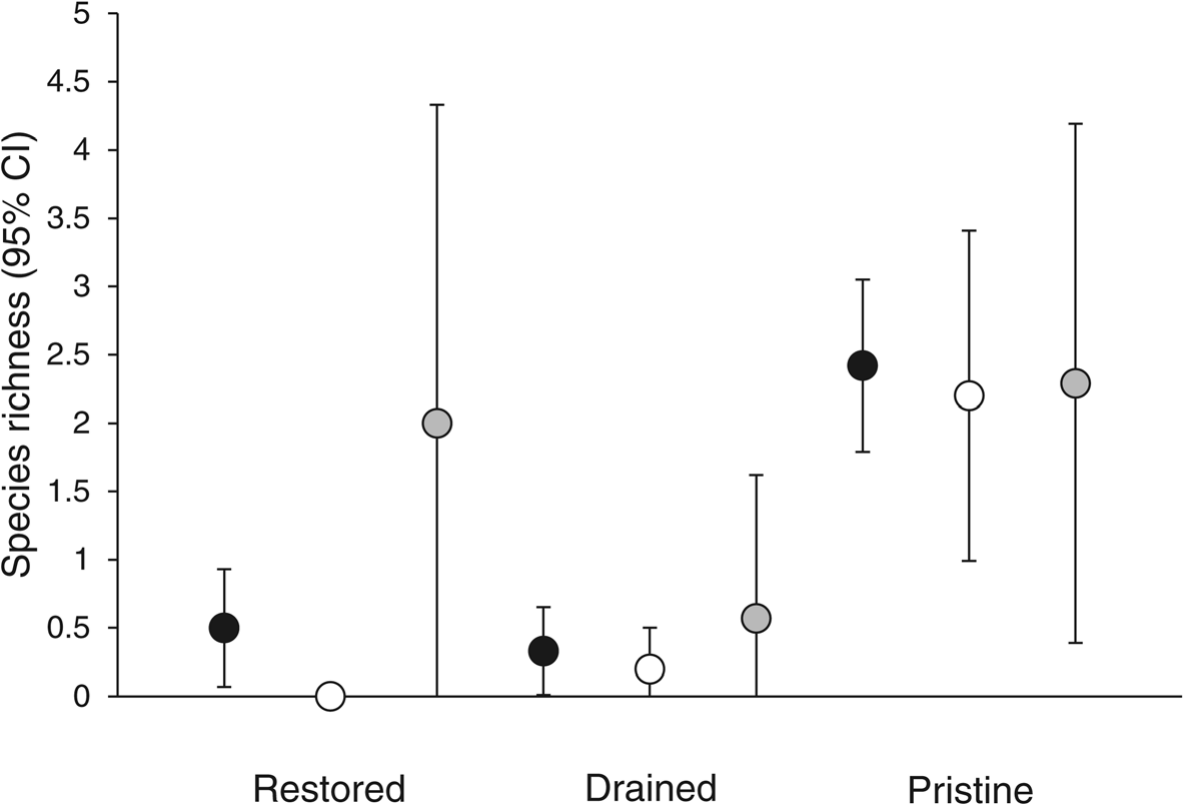
**Odonata monitoring sites.**

The study contained 12 areas (each representing mire complexes of 100′s of hectares) representing geographic variation from 60°52’ N to 64°19’ N and from 21°59’ E to 26°43’ E (Figure 3). From each area there were 3 study sites: i) a pristine site, ii) a drained site, and iii) a drained site which was restored after the first observation. The 12 areas are Natura 2000 sites that include large areas of pristine peatland in the same mire complex. Restoration was conducted inside the Nature 2000 areas and the distance from the restored sites to the closest pristine areas were only a few 10′s of meters. Drainage for forestry was accomplished several decades ago, during 1960s and 1970s. All of the 12 areas were sampled for Odonata during June 2010, before the restoration (two areas were sampled twice before restoration) (for detailed sampling scheme see Additional file 1: Table S1 in Supporting Information). Odonata were sampled as larvae since it confirms the actual breeding of the species and excludes vagrants, in contrast to adults [43]. Moreover, sampling of the larvae is not dependent on the weather conditions. From each site three samples with a water-net with fine mesh size appropriate even for the smallest larvae were taken, each sample representing 2 strong net sweeps. In drained sites samples were taken from ditches and in pristine sites from bog pools. The three samples within each site were taken from a randomly chosen small area of a single ditch or bog pool.

Restorations of different sites were accomplished in different times between November 2010 and March 2014 (Additional file 1: Table S1). Restoration was conducted by filling in the ditches with peat, construction of dams and removal of the tree stands in cases where drainage had increased its growth. After the restoration each area was visited in the first year (end of May - June) after restoration (*n* = 10) and/or in the third year after restoration (*n* = 7). In drained sites the samples were taken from the same ditch and in pristine sites from the same bog pools as before. In the restored sites samples were taken from the newly formed water pool at the site of the previous ditch. Sampling at the first year after restoration was conducted to confirm that there are no larvae (as adult Odonata wouldn’t have time to lay the eggs). Hence, the larvae found in the third year after restoration are due to new colonizations. Larvae were preserved in 70% ethanol and determined to species according to Norling and Sahlén [44]. Specimens which were not identified to species level due to their small size were excluded from the analyses. These specimens represented mainly *Leucorrhinia dubia* and *L. rubicunda* as distinguishing between the two species is dubious in very small larvae.

### Statistical methods

The study set-up represents a nested structure where sites having different treatments are situated within study areas and are thus not independent observations [45]. Moreover, each site was sampled more than once and these observations are not independent either. Thus, we used generalized linear mixed models using Poisson distribution (log link) from the package ‘nlme4’ [46] in R (R Core Team 2014). Abundance or species richness of a site were set as response variables and fixed effects were treatment (restored, drained, pristine), year (before restoration, the first year after restoration, the third year after restoration) and their interaction term denoting the possible effect of restoration. Area and site were added as random factors, and the random effect ‘site’ was nested within the random effect of ‘area’. Abundance and species richness from the three samples within the site were pooled to achieve site level data. For the two areas visited twice before restoration mean number of individuals and species were used. No individuals (and consequently no species) were found from the restored sites during the first year after restoration. This causes inflated standard errors for the parameter in question and thus, we reran the analyses using only the sites sampled before restoration and the third year after restoration (*n* = 7) (Additional file 1: Table S2 in Supporting Information).

Increased sampling of individuals results in increased number of species [42]. Thus, in addition to the real differences in species richness different raw species richness values may occur solely because different number of individuals have been collected, which may in turn reflect important differences in, for instance, resource availability or environmental conditions, but also differences in sampling effort or conditions in sampling [42]. In our case the sampling effort was standardized and thus sampling effort is unlikely to bias the species richness results. Thus, comparison of raw species richness counts here will reflect differences in some environmental conditions between the pristine drained and restored sites. To determine how large proportion of the community we have sampled we created individual-based rarefaction curves i.e. species accumulation curves (SACs) for each site by randomly drawing individuals from the observed species pool 100 times and plotted the mean of these random draws against the number of individuals drawn. When the slope is steep a large fraction of the species is still not sampled and additional sampling would be likely to yield more species and when the slope is shallow it can be concluded that additional sampling would not produce many more species [47]. We used these curves to detect whether the curves i) have reached asymptotes, and (ii) whether there are systematic differences among restored, drained and pristine sites within an area. Even though the data is relatively well replicated the number of observations for each treatment is nevertheless so small that analyzing community differences is not reliable and thus such analyses have not been conducted.

There are no ethical issues that should be considered.

## Additional file

Additional file 1: Table S1. Timing of restoration (R) and sampling before (S0), one year after (S1) and three years after the restoration (S3) of each of the sites. **Table S2.** Results of generalized linear mixed models using only the areas sampled during the third year after restoration. **Figure S1.** Species accumulation curves.
