# Supplementary material for: The effect of peatland drainage and restoration on Odonata species richness and abundance
Source: BMC Ecol. 2015 Apr 9;15:11. doi: 10.1186/s12898-015-0042-z (PMC4412142; doi:10.1186/s12898-015-0042-z)
Supplement: Additional file 1: Table S1. — Timing of restoration (R) and sampling before (S0), one year after (S1) and three years after the restoration (S3) of each of the sites. Table S2. Results of generalized linear mixed models using only the areas sampled during the third year after restoration. Figure S1. Species accumulation curves. [file 12898_2015_42_MOESM1_ESM.pdf]

Table S1. Timing of restoration (R) and sampling before (S0), one year after (S1) and three years after the restoration (S3) of each of the sites

| Name of the site | 2010 | 2011 | 2012 | 2013 | 2014 |
|---|---|---|---|---|---|
| Hirvilamminsuo | S0 | S0, R | S1 | | S3 |
| Kansanneva | S0 | | S0 | R | S1 |
| Kauhaneva | S0 | R | | | S3 |
| Kotkanneva | S0 | R | S1 | | S3 |
| Kuvaja | S0 | | | R | S1 |
| Pilvineva | S0 | | | | R, S1 |
| Pirjatanneva | S0, R | S1 | | S3 | |
| Pitkäsuo | S0 | | | R | S1 |
| Pohjoisneva | S0, R | S1 | | S3 | |
| Salamajärvi | S0 | | R | S1 | |
| Seläntaus I | S0 | R | S1 | | S3 |
| Seläntaus II | S0 | R | | | S3 |

Table S2 Fixed effects (year, treatment, and their interaction term) of generalized linear mixed models for abundance (a) and species richness (b) for areas sampled during the third year after restoration (*n* = 7). Before restoration and pristine sites are used as baselines. Year(3) = third year after restoration, treatment(D) = drained sites, treatment(R) = restored sites. Random variables = study area, study site; number of observations = 42, residual degrees of freedom = 34

| | | Estimate | SE | z | *P* |
|---|---|---|---|---|---|
| a) | Intercept | **2.39** | **0.52** | **4.61** | **<0.001** |
| | year(3) | 0.02 | 0.15 | 0.15 | 0.882 |
| | treatment(D) | **-3.15** | **0.84** | **-3.73** | **<0.001** |
| | treatment(R) | **-4.17** | **0.94** | **-4.43** | **<0.001** |
| | year(3):treatment(D) | 0.45 | 0.43 | 1.04 | 0.297 |
| | year(3):treatment(R) | **2.41** | **0.62** | **3.88** | **<0.001** |
| b) | Intercept | **0.82** | **0.33** | **2.49** | **0.013** |
| | year(3) | -0.12 | 0.34 | -0.34 | 0.732 |
| | treatment(D) | **-1.84** | **0.67** | **-2.76** | **0.006** |
| | treatment(R) | **-2.26** | **0.78** | **-2.88** | **0.004** |
| | year(3):treatment(D) | 0.41 | 0.84 | 0.48 | 0.628 |
| | year(3):treatment(R) | **2.06** | **0.83** | **2.49** | **0.013** |

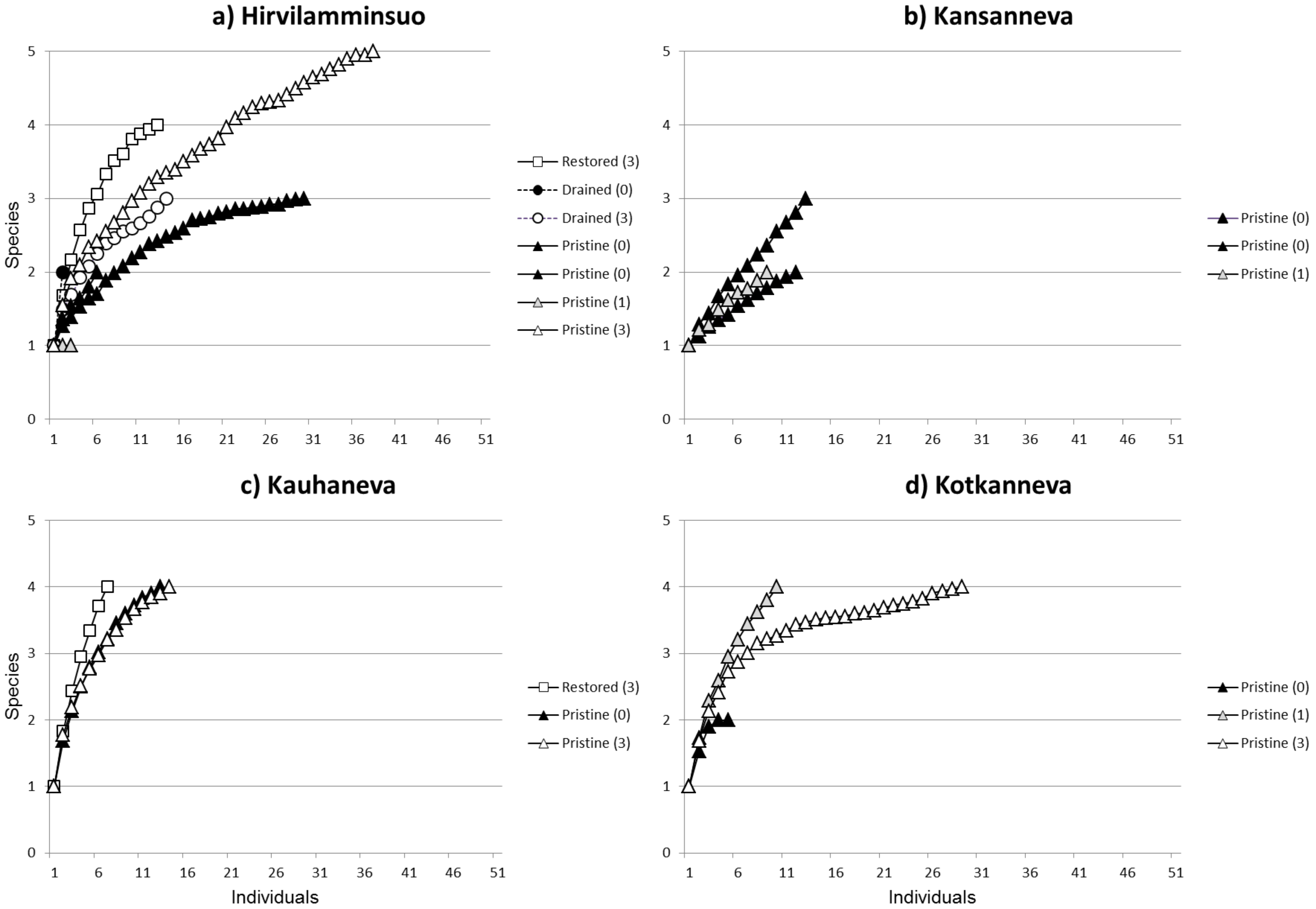
a) Hirvilamminsuo
b) Kansanneva
c) Kauhaneva
d) Kotkanneva
Species
Individuals
Restored (3)
Drained (0)
Drained (3)
Pristine (0)
Pristine (0)
Pristine (1)
Pristine (3)
Pristine (0)
Pristine (0)
Pristine (1)
Restored (3)
Pristine (0)
Pristine (3)
Pristine (0)
Pristine (1)
Pristine (3)

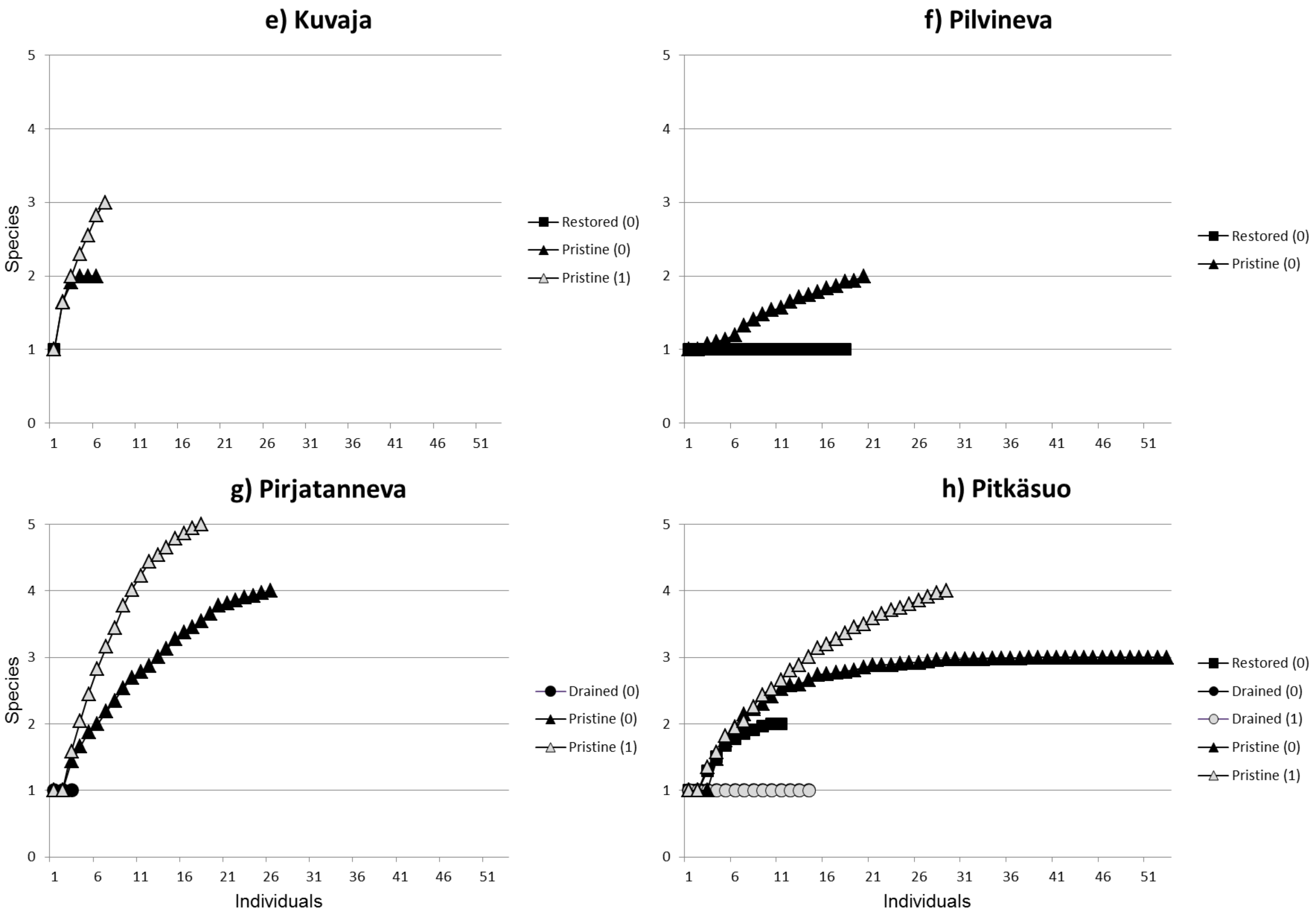

e) Kuvaja
f) Pilvineva
g) Pirjatanneva
h) Pitkäsuo
Species
Individuals
0
1
2
3
4
5
1
6
11
16
21
26
31
36
41
46
51
Restored (0)
Drained (0)
Drained (1)
Pristine (0)
Pristine (1)

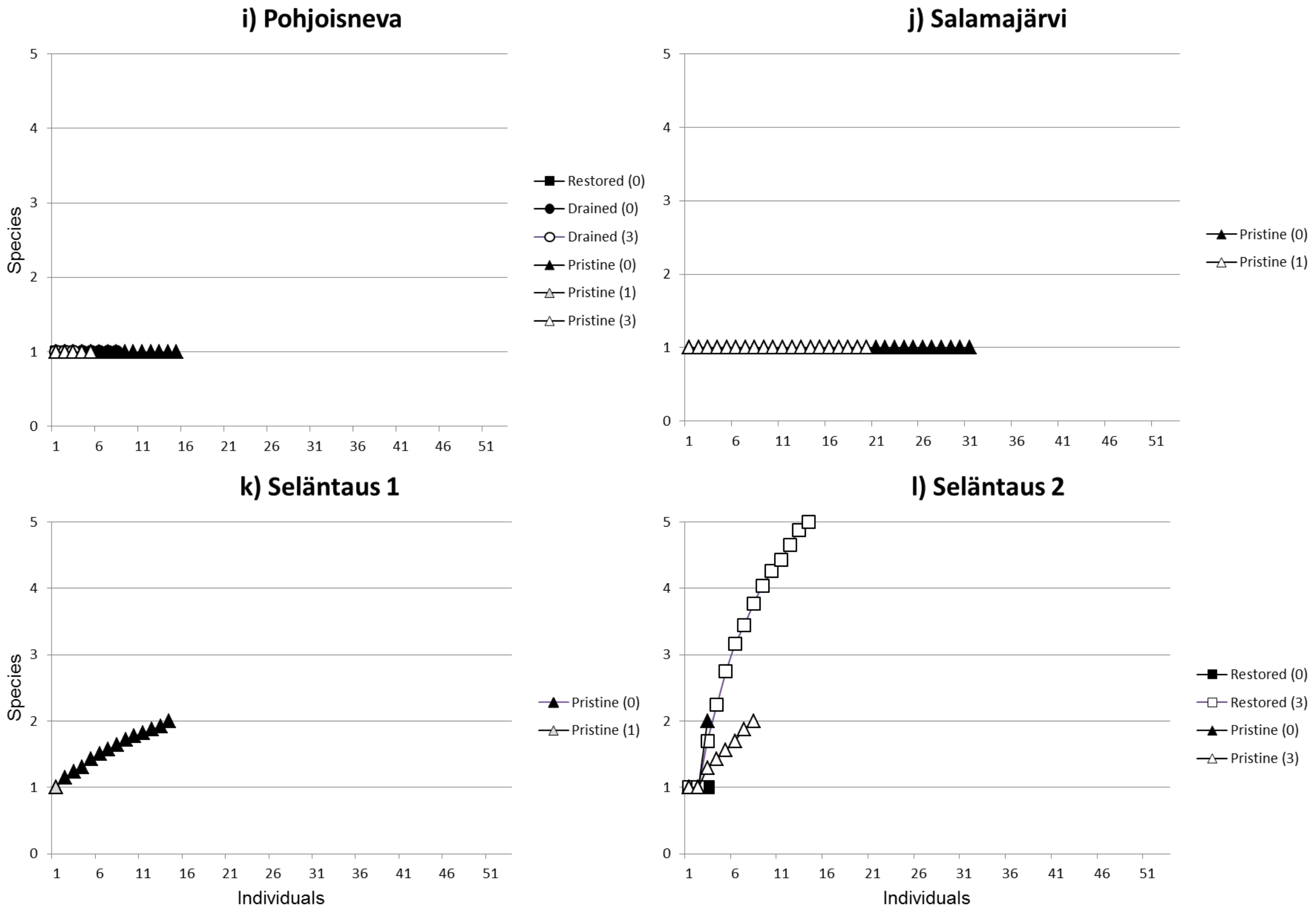


Fig. S1 Species accumulation curves in different study areas (a-l) representing all samplings from which any individuals were found. Legend represent whether the site is Pristine, Restored or Drained and whether the sampling was conducted before restoration (0), ), one year after (1) or three years after the restoration (3)
